# Quantum imploding scalar fields

**DOI:** 10.1098/rsos.180692

**Published:** 2018-10-10

**Authors:** Mark D. Roberts

**Affiliations:** Department of Theoretical Physics, Burpham Institute for Advanced Studies

**Keywords:** singularities, canonical quantum gravity, event horizons

## Abstract

The d’Alembertian □*ϕ* = 0 has the solution *ϕ* = *f*(*v*)/*r*, where *f* is a function of a null coordinate *v*, and this allows creation of a divergent singularity out of nothing. In scalar-Einstein theory a similar situation arises both for the scalar field and also for curvature invariants such as the Ricci scalar. Here what happens in canonical quantum gravity is investigated. Two minispace Hamiltonian systems are set up: extrapolation and approximation of these indicates that the quantum mechanical wave function can be finite at the origin.

## 1. Introduction

For centuries, physicists have wondered what happens at the origin of the reciprocal potential 1/*r*, which is ubiquitous and, for example, occurs in electromagnetism and gravitation. For a minimal scalar field obeying □*ϕ* = 0, the situation is worse because

1.1 $$\phi =\frac{\;f(v)}{r},$$

where *f* is a suitable differentiable function of the null coordinate *v*, a solution allowing creation of a reciprocal singularity out of nothing at the origin of the coordinates *r* = 0. The easiest way to avoid this problem is to say that minimal scalar theory breaks down and another theory is applicable to the problem at hand. There are a huge number of theories to choose from, for example the Born–Infeld theory [1] was created partially to avoid singularities at the origin. In quantum field theory, the scalar field is usually quantized directly so it is hard to compare with the exact solution (1.1). In general, relativity (1.1) was generalized in 1985 [2,3] to a solution of the scalar-Einstein equations: one can have solutions with *ϕ* of the same form but then one needs a compensating null radiation field, if the null radiation field is taken to vanish, then one ends up with a simple scalar-Einstein solution; again one has a scalar field singularity at the origin of the coordinates and there is also a singularity of the space–time curvature, and in this sense the situation is worse than (1.1) because space–time has also broken down. The scalar-Einstein solution has at least six related applications. *Firstly* to cosmic censorship: it is known that in most cases static scalar-Einstein space–times do not have event horizons [4] and the existence of the solution shows that this is also the case in one particular instance in dynamic space–times. Whether event horizons actually exist is now a matter of astrophysical observation [5,6]. *Secondly* to numerical models of gravitational collapse [7,8] where it is a critical value between different behaviours. *Thirdly* to quantum field theories on curved space–times where the scalar field can be equated to the field of the quantum field theory: whether this is an allowable method or not is undecided, in any case it turns out that there are many technical problems concerning whether objects such as the van Vlech determinate converge fast enough. *Fourthly* to the Hawking effect [9], can the exact solution scalar field be equated to scalar fields created in this, a related paper is [10]. *Fifthly* to annihilation and creation operators, perhaps these in some way correspond to imploding and exploding fields; usually these are defined on a fixed background, however as geometry is related to matter there must be a simultaneous change in the gravitational field and perhaps a preferred graviton configuration. *Sixthly* to canonical quantum gravity which is the subject of the present paper.

It is common in physics to let algebraic expressions become functions: however, there is not an established word to describe this. When the algebraic expression is just a constant this is sometimes referred to as letting the object ‘run’, but here sometimes the algebraic expression is a constant times a variable. The words ‘functionify’ and ‘functionification’ do not appear in dictionaries so here the word ‘relax’ is used to describe this process. In the present case what ‘relaxation’ entails is that one component of the metric is taken to be a quantum variable rather than them all, so it is similar to the mini-superspace method in quantum cosmology. Here, the physical motivation is that the system is described by two variables, the scalar field and the killing potential, each of which in turn is relaxed. In other words, the two classically descriptive variables are replaced in turn by quantum ones.

Section 2 describes the properties of the scalar-Einstein solution needed here, in particular the original single null and double null forms are presented, brute force methods applied to these forms leads to two variable problems. The solution has two characteristic scalars: the scalar field and the homothetic Killing potential, and expressing the solution in terms of these leads to one variable problems. Section 3 describes how to get a Hamiltonian and quantize the system when the homothetic Killing vector is relaxed. This can be pictured as what happens when there is one quantum degree of freedom introduced into the system corresponding to fluctuations in the homothetic Killing vector away from its classical properties; classical fluctuation have been discussed by Frolov [11]. Section 4 describes how to get a Hamiltonian and quantize when the scalar field is relaxed. Section 5 discusses how to fit the results of the previous two sections together and many of the assumptions of the model. Section 6 discusses speculative applications and concludes. Conventions used are signature − + + +, indices and arguments of functions left out when the ellipsis is clear, *V* to describe a scalar field potential and *U* to describe the Wheeler–DeWitt potential, *ϕ* for the scalar field in a scalar-Einstein solution, *ξ* for a source scalar field, field equations $G_{\mu \nu }=G_{\mu \nu }-8\pi \kappa T_{\mu \nu }\;\mu ,\nu ,\ldots$ are space–time coordinates, *A*, *B*, … are field variables.

## 2. The scalar-Einstein solution

The solution in the original single null coordinates [2,3,12] is

2.1 $$\left.\begin{array}{rl} \text{d}s^{2} & =-(1+2\sigma )\;\text{d}v^{2}+2\;\text{d}v\;\text{d}r+r(r-2\sigma v)\;\text{d}\Sigma_{2}^{2}\\ \;\text{and}\;\text{d}\Sigma_{2}^{2} & \equiv \text{d}\theta^{2}+\sin(\theta )\;\text{d}\phi^{2},\;\phi =\frac{1}{2}\ln\left(1-\frac{2\sigma v}{r}\right), \end{array}\right\}$$

the Ricci scalar is given by

2.2 $$R=\frac{2\sigma^{2}v}{r^{2}{\left(r-2\sigma v\right)}^{2}}((1+2\sigma )v-2u),$$

with other curvature invariants such as the Riemann and Weyl tensors squared being simple functions of it. The homothetic Killing vector is

2.3 $$K=Cv(2r+(1-2\sigma )v),\;K_{a}K^{a}=-4CK,\;K_{;ab}=-2Cg_{ab},$$

with conformal factor −2*C*. Defining the null coordinate

2.4 u≡(1+2σ)v−2r,

the solution takes the double null form

2.5 $$\left.\begin{array}{rl} \text{d}s^{2} & =-\text{d}u\;\text{d}v+r_{+}r_{-}\text{d}\Sigma_{2}^{2},\;\text{d}\Sigma_{2}^{2}=\text{d}\theta^{2}+\sin{\left(\theta \right)}^{2}\;\text{d}\phi^{2}\\ \;\text{and}\;r_{\pm } & =(1\pm 2\sigma )v-u,\;\phi =\frac{1}{2}\ln\left(\frac{r_{-}}{r_{+}}\right),\;K=Cuv,\;R=\frac{2\sigma uv}{r_{+}^{2}r_{-}^{2}} \end{array}\right\}$$

To transform the line element to a form in which the scalar field and homothetic Killing potential are coordinates define

2.6 $$y\equiv \frac{K}{C}=uv,\;v^{2}=\frac{y}{1+2\sigma f(x)},\;u^{2}=y(1+2\sigma f(x)),$$

*f* = coth gives the region *uv* > 0

2.7 $$\text{d}s^{2}=-\frac{\text{d}y^{2}}{4y}+\frac{\sigma^{2}y}{sl{\left(x\right)}^{2}}\;\text{d}x^{2}+\frac{\sigma^{2}y}{sl(x)}\;\text{d}\Sigma_{2}^{2},\;x=\phi ,\;R=2g^{xx},$$

*f* = tanh gives the region *uv* < 0

2.8 $$\text{d}s^{2}=-\frac{\sigma^{2}y}{cl{\left(x\right)}^{2}}\;\text{d}x^{2}+\frac{\text{d}y^{2}}{4y}+\frac{\sigma^{2}y}{cl(x)}\;\text{d}\Sigma_{2}^{2},$$

where the functions *sl* and *cl* are given by

2.9 $$\left.\begin{array}{rl} sl(x) & \equiv \sinh(x)(\sinh(x)+2\sigma \cosh(x))\\ \;\text{and}\;cl(x) & \equiv \cosh(x)(\cosh(x)+2\sigma \sinh(x)), \end{array}\right\}$$

with the properties

2.10 $$\left.\begin{array}{rl} cl-sl & =1,\;sl^{\prime \prime }=4sl+2,\;cl^{\prime \prime }=4cl-2,\\ sl^{\prime } & =2\sqrt{sl^{2}+sl+\sigma^{2}},\;\;cl^{\prime }=2\sqrt{cl^{2}-cl+\sigma^{2}},\\ \;\text{and}\;sl(\phi ) & =\frac{4\sigma^{2}uv}{r_{+}r_{-}}=2\sqrt{2\sigma^{3}yR},\;cl(\phi )=\frac{1}{r_{+}r_{-}}(v-u)((1-4\sigma^{2})v-u). \end{array}\right\}$$

**Figure 1.**
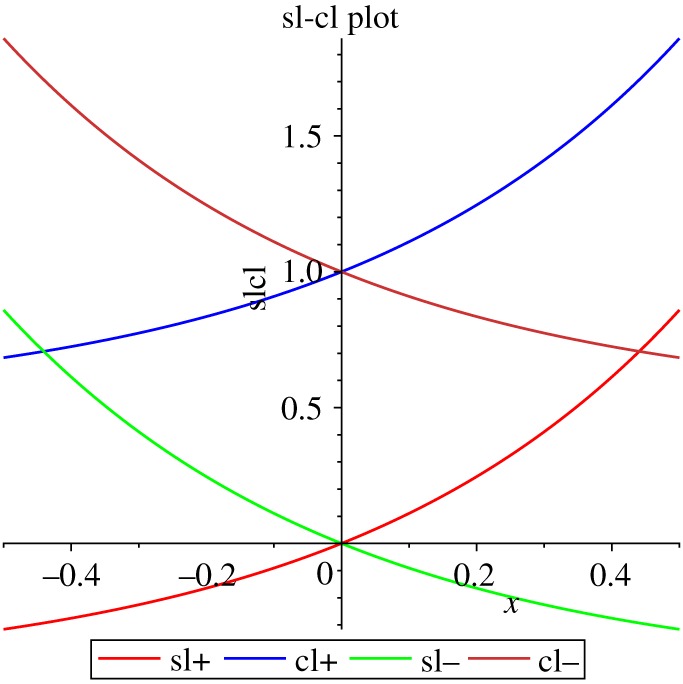
sl and cl, $\sigma =\pm \frac{1}{2}$.

Properties of this solution such as junction conditions have recently been discussed [12] (figure 1).

## 3. Relaxation of the homothetic Killing potential

Consider the line element (2.7), let *y* = *t*^2^ then relax *σt* to become a ‘scale factor’ function *a*(*t*)

3.1 $$\text{d}s^{2}=-\text{d}t^{2}+a{\left(t\right)}^{2}\left[\frac{\text{d}x^{2}}{sl{\left(x\right)}^{2}}+\frac{\text{d}\Sigma_{2}^{2}}{sl(x)}\right],$$

which is similar to the Robertson–Walker line element, the difference being that (3.1) involves the function *sl*(*x*), defined in (2.9). Scalar-Einstein Robertson–Walker solutions have been discussed in [13] and their quantum cosmology in [14]. Couple the line element (3.1) to the source

3.2 $$R_{\mu \nu }=2\xi_{\mu }\xi_{\nu }+g_{\mu \nu }V_{1}(\xi ),$$

to form field equations $G^{ab}$ which is the Einstein tensor with the source subtracted off. Having $G_{x}^{t}$ necessitates *ξ*_*t*_ξ_*x*_ = 0 take *ξ*_*t*_ = 0; *ξ*_*x*_ = 1 is forced by the requirement that $G_{b}^{a}$ is independent of *x*. After using the differential properties of *sl*, see (2.10) the field equations become

3.3 $$-a^{2}G_{t}^{t}=3{\dot{a}}^{2}-3\sigma^{2}+a^{2}V_{1},\;-a^{2}G_{x}^{x}=-a^{2}G_{\theta }^{\theta }=2a\ddot{a}+{\dot{a}}^{2}-\sigma^{2}+a^{2}V_{1}.$$

The momentum and Hamiltonian can be read off

3.4 $$\pi_{a}=3a\dot{a},\;H_{1}=\frac{\pi_{a}^{2}}{6a}+U_{1}=-\frac{a^{3}}{2}G_{t}^{t},\;U_{1}\equiv -\frac{3}{2}\sigma a+\frac{1}{2}a^{3}V_{1}.$$

The *q* Hamiltonian equation is immediate, the *π* Hamiltonian equation is

3.5 $${\dot{\pi }}_{a}+\frac{\partial H_{1}}{\partial a}=-\frac{3a^{2}}{2}G_{x}^{x}.$$

The mini-metric is

3.6 $$M_{1}^{aa}=\frac{1}{6a},\;\det(M_{1})=6a,$$

which has vanishing mini-curvature. Using the quantization substitution

3.7 $$\pi_{A}\to -ı\hbar \nabla_{A}$$

so that the Hamiltonian (3.4) becomes the Wheeler [15]–DeWitt [16] equation

3.8 $$H_{1}\psi =-\frac{\hbar^{2}}{6a}◻\psi +U\psi .$$

Using the mini-metric (3.6)

3.9 $$◻\psi =\frac{1}{\sqrt{6a}}{\left(\frac{\psi_{a}}{\sqrt{6a}}\right)}_{,a},$$

the Hamiltonian (3.8) becomes

3.10 $$-\frac{72a^{3}}{\hbar^{2}}H_{1}\psi =2a\psi_{,aa}-\psi_{,a}+\frac{36a^{4}}{\hbar^{2}}(3\sigma^{2}-a^{2}V_{1})\psi .$$

For *V*_1_ = 0 maple finds a solution that is a linear combination of Bessel functions $B_{Y}^{J}$

**Figure 2.**
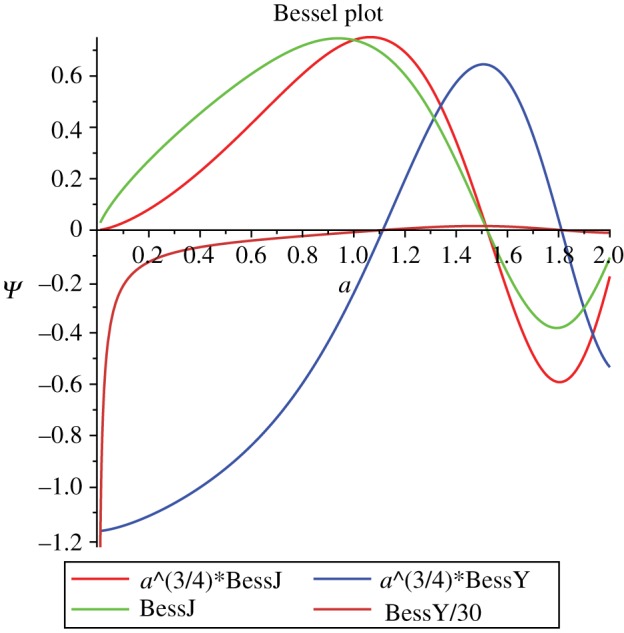
Bessel functions for a, $\sigma =5\hbar /(6\sqrt{(}6))$.

3.11 $$\psi_{1}=\sum_{JY}C_{Y}^{J}a^{3/4}B_{Y}^{J}\left(\frac{3}{10},\frac{6\sqrt{6}\sigma }{5\hbar }a^{5/2}\right),$$

where $C_{Y}^{J}$ are amplitude constants. These Bessel function are illustrated in the second figure 2. Expanding (3.11) for small *a*

3.12 $$\begin{array}{rl} \psi_{1} & =-\frac{2^{3/10}C_{Y}}{\Gamma (7/10)\sin(3\pi /10)}\\ & \;+\frac{5}{3}2^{7/10}\Gamma \left(\frac{7}{10}\right)\left(\sin\left(\frac{3\pi }{10}\right)C_{J}+\cos\left(\frac{3\pi }{10}\right)C_{Y}\right)a^{3/2}+O(a^{5})\\ & \;\approx 1.17C_{Y}+(2.84C_{J}+2.07C_{Y})a^{3/2}, \end{array}$$

so that in particular the limit as *a* → 0 is given by the finite value of the first term of (3.12).

In the asymptotically flat region, one wants the wave function to vanish. By inspection, one can see that this is the case, for $\sigma =+\frac{1}{2}$, *sl*(0) = 1 so that one only has to consider the large *a* value of the Bessel functions; from the second figure 2 it can be seen that it decreases in value and oscillates around zero. Explicitly, expanding the BesselJ function around *a* = infinity

3.13 $$ABJ=6^{-}\frac{3}{4}\sqrt{\frac{10\hbar }{\pi \sigma }}\sin\left(\frac{6\sqrt{6}\sigma }{5}a^{5/2}+\frac{\pi }{10}\right)\frac{1}{\sqrt{a}}+O(z^{-3}),$$

which indeed oscillates fast and decays as *a*^−1/2^.

To normalize the wave function the standard procedure is

3.14 $$1=\int_{-\infty }^{\infty }\psi \psi^{*}\text{d}a,$$

where the lower limit is needed as *a* can take negative values. If this is followed then the indefinite integrals can be computed and maple gives hypogeometric functions, however the infinite limits of these are not computable by maple. Restricting attention to the BesselJ term, if one replaces the *a*^3/4^ premultiplicative term in the wave function (3.11) by *a*^3/4^ + *ε*, then the limits of the integral are computable and fix *C*_*J*_ by

3.15 $$\frac{1}{C_{J}^{2}}=\frac{1}{36\sqrt{\pi }}6^{1/2-6\epsilon /5}5^{4\epsilon /5}\frac{\Gamma (\left(4+2\epsilon \right)/5)\Gamma \;(-\left(2\epsilon \right)/5)}{\Gamma (\left(4-2\epsilon \right)/5)\Gamma (1/2-2\epsilon /5)}{\left(\frac{\sigma }{\hbar }\right)}^{-1-4\epsilon /5}\{1-{\left(-1\right)}^{2\epsilon }{ı}^{-4\epsilon /5}\},$$

for *ε* = 0 the curly bracket term vanishes giving infinite *C*_*J*_, but for *ε* ≠ 0 it is finite and in this sense the wave function is ‘almost’ normalizable.

## 4. Relaxation of the scalar field

Relaxing the scalar field in (2.8) gives line element

4.1 $$\text{d}s^{2}=-\sigma^{2}y\beta {\left(t\right)}^{4}\;\text{d}t^{2}+\frac{\text{d}y^{2}}{4y}+\sigma^{2}y\beta {\left(t\right)}^{2}\;\text{d}\Sigma_{2}^{2},$$

*y* remains a homothetic Killing potential, obeying the last two equations of (2.3), regardless of the choice of *β*; $\beta =1/\sqrt{cl}$ recovers the scalar-Einstein solution (2.8), this choice of power of *β* is made for later convenience. After subtracting off the source, *V*_2_ has to vanish or else *y* is manifest. The field equations become

4.2 $$\left.\begin{array}{rl} -\sigma^{2}y\beta^{6}G_{.t}^{t} & ={\dot{\beta }}^{2}+\beta^{4}-\sigma^{2}\beta^{6}-\beta^{2}{\dot{\xi }}^{2}\\ \;\text{and}\;+\sigma^{2}y\beta^{6}G_{.\theta }^{\theta } & =-\beta \ddot{\beta }+2{\dot{\beta }}^{2}+\sigma^{2}\beta^{6}-\beta^{2}{\dot{\xi }}^{2},\;G_{.r}^{r}=2G_{.\theta }^{\theta }+G_{.t}^{t}. \end{array}\right\}$$

The momenta are

4.3 $$\pi_{\beta }=\frac{2\sigma \dot{\beta }}{\beta^{2}},\;\pi_{\xi }=-2\sigma \dot{\xi }.$$

The Hamiltonian is

4.4 $$H_{2}=\frac{\beta^{2}}{4\sigma }\pi_{\beta }^{2}-\frac{1}{4\sigma }\pi_{\xi }^{2}+U_{2}=-\sigma^{3}y\beta^{4}G_{.t}^{t},\;U_{2}=\sigma \beta^{2}(1-\sigma^{2}\beta^{2}),$$

and the *π_β_* Hamilton equation is

4.5 $${\dot{\pi }}_{\beta }+\frac{\partial H_{2}}{\partial \beta }=-2\sigma^{3}y\beta^{3}(G_{.\theta }^{\theta }+G_{.t}^{t}).\;$$

The mini-metric is

4.6 $$M_{2AB}=\left(\begin{array}{cc} \frac{4\sigma }{\beta^{2}} & 0\\ 0 & -4\sigma \end{array}\right),\;\;\sqrt{-\det(M_{2})}=\frac{4\sigma }{\beta }.$$

As before using (3.7) gives the Wheeler–DeWitt equation

4.7 $$\frac{4\sigma }{\hbar^{2}}H_{2}\psi =-\beta {\left(\beta \psi_{\beta }\right)}_{\beta }+\psi_{\xi \xi }+\frac{4\sigma^{2}\beta^{2}}{\hbar^{2}}(1-\sigma^{2}\beta^{2})\psi ,$$

with solution

4.8 $$\psi_{2}=\frac{1}{\beta }\sum_{+-}A_{-}^{+}\exp\left(\frac{\pm \varepsilon \xi }{\hbar }\right)\sum_{\mathrm{MW}}C_{W}^{M}W_{W}^{M}\left(\frac{ı}{2\hbar },\frac{\varepsilon }{2\hbar },\frac{2ı\sigma^{2}\beta^{2}}{\hbar }\right),$$

where *A*_+_, *A*_−_, *C*_M_, *C*_W_ are complex amplitude constants and *ɛ* is a non-negative real source scalar field constant; there is a qualitative difference between *ɛ* = 0 and *ɛ* ≠ 0, the former jumps at *β* = 0 and the latter does not: only *ψψ*^†^ is measurable and for that there is no jump in either case. Taking $1=2\sigma =\hbar =2A_{+}=2A_{-}=C_{\text{M}},0=C_{\text{W}}$ and expanding the WhittakerM function for small *β*

4.9 $$\psi_{W}=\frac{1}{2}\left[\beta +\frac{\beta^{3}}{8}+O(\beta^{5})\right],$$

expanding the exponential term for small *ξ*

4.10 $$\psi_{e}=\left[1+\frac{\xi^{2}}{2}+\frac{\xi^{4}}{24}+O(\xi^{5})\right],$$

expanding all (4.8) to lowest order

4.11 $$\psi_{2}=k^{\prime }\beta^{\varepsilon },$$

where *k*′ is a complex constant which varies for different *ɛ*.

**Figure 3.**
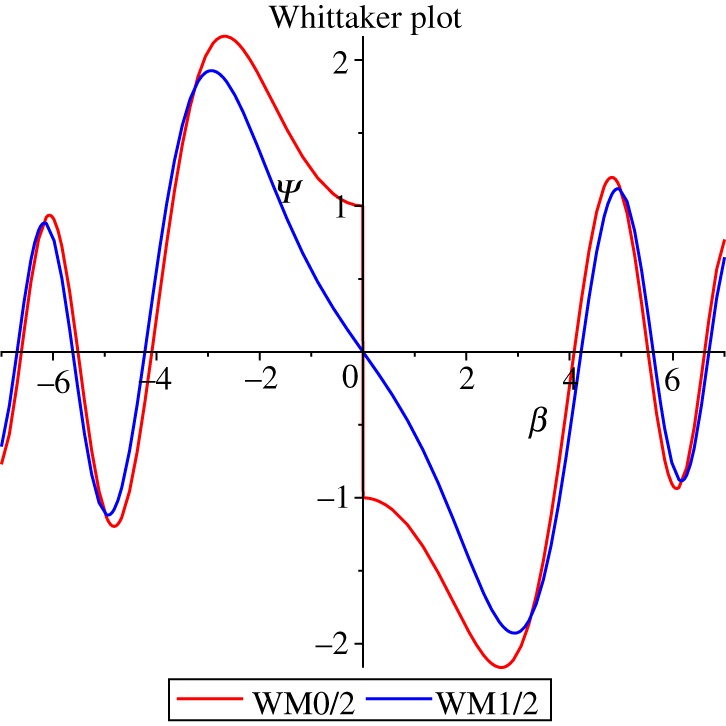
$1=2\sigma =\hbar =C_{\text{M}},\;0=C_{\text{W}}$, red $\left(ı-1)W_{\text{M}}(\varepsilon =0\right)$, blue $ıW_{\text{M}}(\varepsilon =1)$.

In the asymptotically flat region *β* → 0 so if one chooses the blue Whittaker function in figure 3 and the negative exponential in (4.8) the wave function also vanishes in the asymptotically flat region. Normalization using (3.14) is not maple computable because the Whittaker functions do not integrate.

## 5. Extrapolation, approximation and generalization

Extrapolation by combining §§3 and 4 gives the wave function to lowest order

5.1 $$\psi =k^{\prime }a^{\frac{3}{2}}\beta^{\varepsilon },$$

where *k*′ is a complex constant, transferring to double null coordinates using (2.10) gives

5.2 $$\psi =k{\left(uv\right)}^{3/4}{\left[\frac{r_{+}r_{-}}{\left(v-u)((1-4\sigma^{2})v-u\right)}\right]}^{\varepsilon /2},$$

where *k* is a complex constant. The singularity is at *u* = (1 ± 2σ) *v* where the wave function takes the form

5.3 $$\psi {|}_{\pm }=k{\left(1\;\pm \;2\sigma \right)}^{3/4}v^{3/2}{\left[\frac{\mp r_{\pm }}{\sigma (1\;\pm \;2\sigma )v}\right]}^{\varepsilon /2},$$

substituting *r*_±_ for *ɛ* > 0 the wave function vanishes at the singularity: the desired result. For *ɛ* = 0 the wave function is a simple function of *v*, for *ɛ* < 0 the Whittaker functions (4.8) are not defined.

There are several assumptions used in arriving at (5.2). *Firstly*, it has been assumed that a wave function derived in one segment of the space time can be extended to the whole space time, in particular *v* = 0 and *u* = 0 regions are not included in the coordinate systems (2.7) and (2.8) and these regions are needed if one wants to study junctions with flat space time, however, the curvature singularity exists in both systems in the same sense: in the double null form (2.5) the curvature singularity is at *g*^*θθ*^ and the line element truncates here, similarly for (2.7) and (2.8) at *g*^*xx*^; and in this sense, the wave function exists at the classically singular point. The Aharanov–Bohm [17,18] not only shows the existence of the vector potential, it also shows that the wave function is smooth rather than discontinuous at boundaries, and this justifies the preference of a smooth wave function here. *Secondly*, no boundary conditions on the quantum system are applied; these would cut down on the large number of constants in the solutions (3.11) and (4.8) and for the present purposes are unlikely to make a difference as we require existence not uniqueness. *Thirdly*, no method of extracting information from the wave function has been given, so there is no method of recovering the curvature singularity from it: it might happen that any such method must itself be in some sense singular. *Fourthly*, the wave functions in the two regions can be combined and furthermore done so without considerations of phase. For large distances, the wave functions (3.11) and (4.8) are approximately trigonometric but it is not clear whether they peak and dip at the same time or not. The Hamiltonian, which is a linear combination of (3.4) and (4.4), has a separable solution which is a product of (3.11) and (4.8); explicitly $4\sigma (H_{\beta }\psi +\ell H_{a}\psi )/\hbar^{2}$ has the solution

5.4 $$\psi =C\frac{a^{3/4}}{\beta }\cosh\left(\frac{\varepsilon \xi }{\hbar }\right)BJ\left(\frac{3}{10},\frac{6\sqrt{6}\sigma }{5\hbar }a^{5/2}\right)WM\left(\frac{ı}{2\hbar },\frac{\varepsilon }{2\hbar },\frac{2ı\sigma^{2}\beta^{2}}{\hbar }\right),$$

where C is an amplitude constant, note (5.4) is independent of *ℓ*.

## 6. Conclusion

The above systems $H_{1},H_{2}$ are not restricted to be either exploding or imploding: such restrictions might come from additional physical assumptions. The particle content corresponding to the above wave picture is not clear; it is not even clear if it at best corresponds to one or many particles. Presumably, the content of a scalar field is so configured that it cancels out the energy of gravitons, giving no overall energy which would agree with the classical case. For microscopic application to annihilation and creation operators, the above Hamiltonians $H_{1},H_{2}$ could be the first step in finding out how space time changes. For macroscopic application to ‘black holes’ and ‘white holes’, again the Hamiltonians could be a first step in solving the ‘back reaction’ problem.

Our conclusion is that in the specific case studied here where classical space time has curvature singularities the quantum mechanical wave function can be finite, and that furthermore this could be an indication of general behaviour.

## Data accessibility

There are no data associated with this paper; it should be replicable by anyone with pen and paper and 300 h to do calculations.
